# Hsp90 Interacts with the Bacterial Effector NleH1

**DOI:** 10.3390/pathogens7040087

**Published:** 2018-11-13

**Authors:** Miaomiao Wu, Philip R. Hardwidge

**Affiliations:** Department of Diagnostic Medicine/Pathobiology, Kansas State University, Manhattan, KS 66506, USA; miaomiaowu@ksu.edu

**Keywords:** *E. coli*, Hsp90, NleH1, RPS3

## Abstract

Enterohemorrhagic *Escherichia coli* (EHEC) utilizes a type III secretion system (T3SS) to inject effector proteins into host cells. The EHEC NleH1 effector inhibits the nuclear factor kappa-light-chain-enhancer of activated B cells (NF-κB) pathway by reducing the nuclear translocation of the ribosomal protein S3 (RPS3). NleH1 prevents RPS3 phosphorylation by the IκB kinase-β (IKKβ). IKKβ is a central kinase in the NF-κB pathway, yet NleH1 only restricts the phosphorylation of a subset of the IKKβ substrates. We hypothesized that a protein cofactor might dictate this inhibitory specificity. We determined that heat shock protein 90 (Hsp90) interacts with both IKKβ and NleH1 and that inhibiting Hsp90 activity reduces RPS3 nuclear translocation.

## 1. Introduction

The nuclear factor kappa-light-chain-enhancer of activated B cells (NF-κB) family of transcription factors regulates innate and adaptive immune responses. In addition to the well-characterized Rel family proteins [1], ribosomal protein S3 (RPS3) is a key non-Rel subunit and was identified as a “specifier” NF-κB component. RPS3 guides NF-κB to specific κB sites by increasing the affinity of the NF-κB p65 subunit for target gene promoters [2]. Activation of NF-κB signaling is initiated by external stimuli that activate the IκB kinase (IKK) complex. This complex consists of two catalytic components, IKKα (IKK1) and IKKβ (IKK2), in addition to a regulatory subunit, the NF-κB Essential Modulator (NEMO; IKKγ). The canonical IKK complex plays a central role in regulating many cellular processes in response to a variety of physiological and pathological stimuli, among which NF-κB is the best known. Activated IKKβ phosphorylates IκBα, resulting in its subsequent ubiquitination and degradation, which allows for p65 and p50 nuclear translocation [3]. IKKβ also phosphorylates RPS3 on Ser209, enhancing its association with importin-α and mediating RPS3 nuclear translocation [4].

Bacteria have evolved secretion systems to transport virulence proteins, termed ‘effectors’, to counteract host innate immunity. Enterohemorrhagic *Escherichia coli* (EHEC) encodes numerous type three secretion system (T3SS) effectors that subvert cellular processes to create an environment conductive to bacterial survival. EHEC encodes two forms of the NleH effector, NleH1 and NleH2 [5,6]. Both effectors bind RPS3, but only NleH1 inhibits RPS3 nuclear translocation by preventing RPS3 phosphorylation on S209 by IKKβ [4,5]. NleH1 activity only restricts the phosphorylation of a subset of the IKKβ substrates, suggesting that an additional host co-factor might dictate this inhibitory specificity. Here we identified heat shock protein 90 (Hsp90) as an NleH1 binding partner and found that inhibiting Hsp90 activity reduced RPS3 nuclear translocation.

## 2. Results

### 2.1. NleH1 and NleH2 Bind to IKKβ

**Figure 1:**
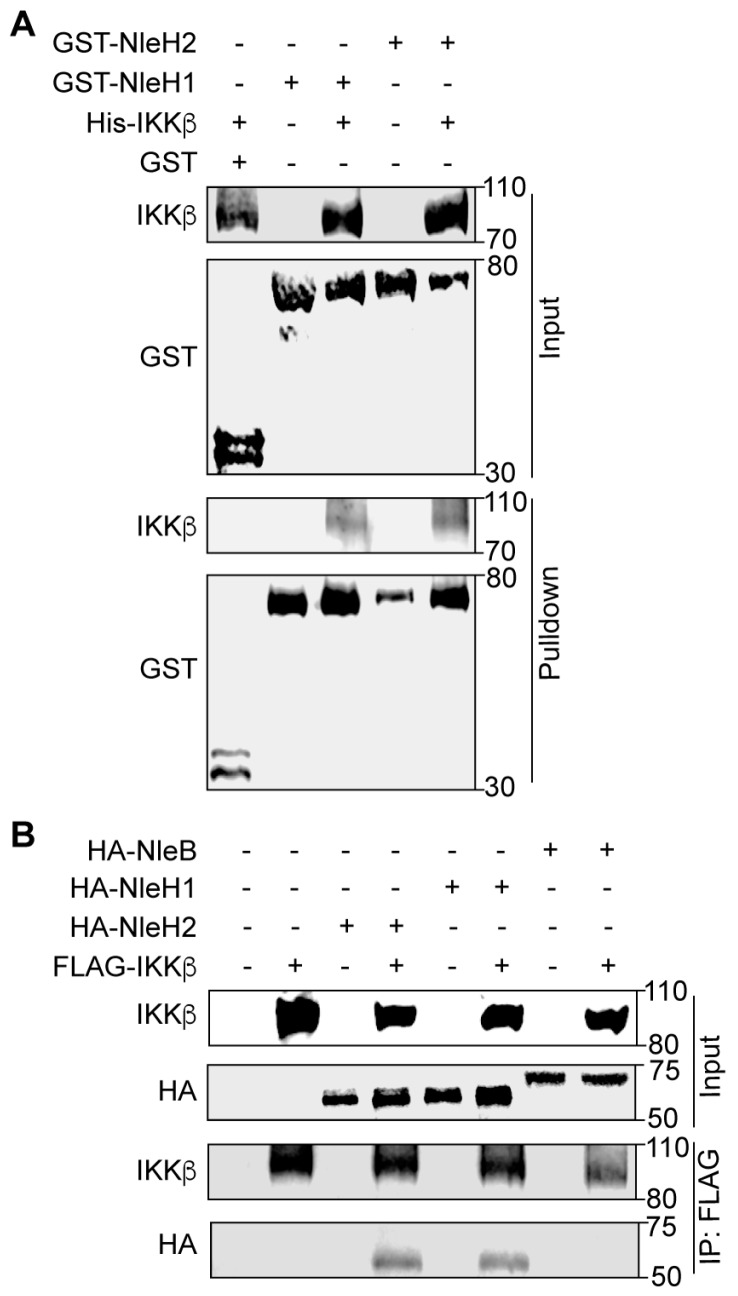
NleH1 and NleH2 bind to IKKβ. (**A**) Pulldown assay to detect binding between NleH1, NleH2, and IKKβ. His6-IKKβ was incubated with GST, GST-NleH1 and GST-NleH2 and subjected to a GST pulldown assay using glutathione-sepharose beads. Protein complexes were eluted with 10 mM reduced glutathione followed by 10% SDS-PAGE analysis. GST was used as a negative control. (**B**) Immunoprecipitation of NleB1-HA, NleH1-HA and NleH2-HA with FLAG-IKKβ. HEK293 cells were transfected and cell lysates were immunoprecipitated using anti-FLAG M2 gel and immunoblotted for FLAG and HA. NleB1-HA was used as a negative control.

To determine whether EHEC NleH1 binds directly to IKKβ we conducted GST pulldown assays. His-IKKβ, GST, GST-NleH1 and GST-NleH2 were purified using nickel-nitrilotriacetic acid (Ni-NTA) agarose beads. Purified GST (negative control), GST-NleH1 and GST-NleH2 were immobilized on GST beads and incubated with His-IKKβ. IKKβ was enriched in the NleH1 and NleH2 pulldown samples, as compared to the negative control (Figure 1A). To determine whether NleH1 and NleH2 interact with IKKβ in mammalian cells, we performed co-immunoprecipitation experiments. After co-transfecting either NleB1-HA (as a negative control), NleH1-HA or NleH2-HA with FLAG-IKKβ, cell lysates were immunoprecipitated with anti-FLAG M2 beads and subsequently immunoblotted. NleH1-HA and NleH2-HA, but not NleB1-HA, interacted with FLAG-IKKβ (Figure 1B). Thus, NleH1 and NleH2 bind directly to IKKβ in vitro, and interact with IKKβ in mammalian cells.

To identify other proteins that are enriched with IKKβ as a function of co-expression of NleH1, we repeated the transfection experiments described above and then processed the immunoprecipitated proteins samples for mass spectrometry analysis. We identified Hsp90, Cdc37 and RPS3 in these samples.

Hsp90 is an important chaperone protein that regulates the folding, stability and trafficking of many client proteins [7]. Cell division cycle protein 37 (Cdc37) is one of the best-studied Hsp90 co-chaperones [8,9]. Hsp90 interacts with Cdc37 and the Hsp90-Cdc37 complex can form a stable complex with IKK, which plays a critical role in its activation and regulation [10,11]. Due to the important association between Hsp90 and IKK, we focused on the potential interactions between Hsp90, IKKβ, and NleH1.

### 2.2. Hsp90 Interacts with NleH1, NleH2 and IKKβ

**Figure 2:**
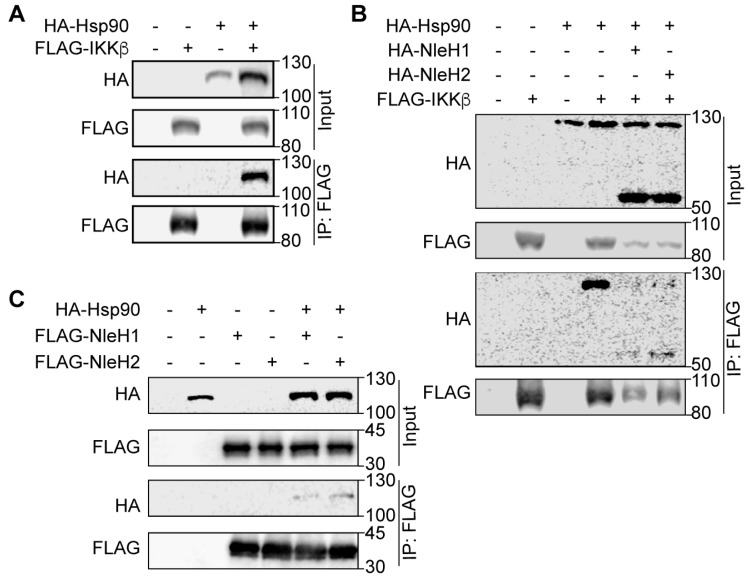
Interaction of NleH1, Hsp90 and IKKβ. (**A**) Immunoprecipitation of Hsp90-HA with FLAG-IKKβ. HEK293 cells were transfected with FLAG-IKKβ and Hsp90-HA. Cell lysates were immunoprecipitated using anti-FLAG M2 gel and immunoblotted for FLAG and HA. (**B**) Immunoprecipitation of NleH1-HA/NleH2-HA and Hsp90-HA with FLAG-IKKβ. HEK293 cells were transfected with FLAG-IKKβ Hsp90-HA, NleH1-HA and NleH2-HA. Cell lysates were immunoprecipitated using anti-FLAG M2 gel and immunoblotted for FLAG and HA. (**C**) Immunoprecipitation of Hsp90-HA with FLAG-NleH1 and FLAG-NleH2. HEK293 cells were transfected with FLAG-NleH1, FLAG-NleH2 and Hsp90-HA. Cell lysates were immunoprecipitated using anti-FLAG M2 gel and immunoblotted for FLAG and HA.

To assess the association of Hsp90 and IKKβ in mammalian cells, we performed co-transfection and co-immunoprecipitation experiments with Hsp90-HA and FLAG-IKKβ. HEK293 cell lysates were immunoprecipitated with anti-FLAG M2 beads and subsequently immunoblotted with anti-FLAG and anti-HA antibodies. Hsp90-HA interacted with FLAG-IKKβ (Figure 2A), which is consistent with a previous study showing that endogenous Hsp90 co-precipitated from HeLa cell extracts with IKKβ [10]. We considered whether IKKβ might interact with both NleH1/NleH2 and Hsp90. To test this idea, we co-transfected HEK293 cells with FLAG-IKKβ Hsp90-HA and NleH-HA. We observed that IKKβ interacted with Hsp90, NleH1 and NleH2 (Figure 2B) and that Hsp90 interacted with both NleH1 and NleH2 (Figure 2C).

### 2.3. Hsp90 Is a Cofactor of Both NleH1 and IKKβ

**Figure 3:**
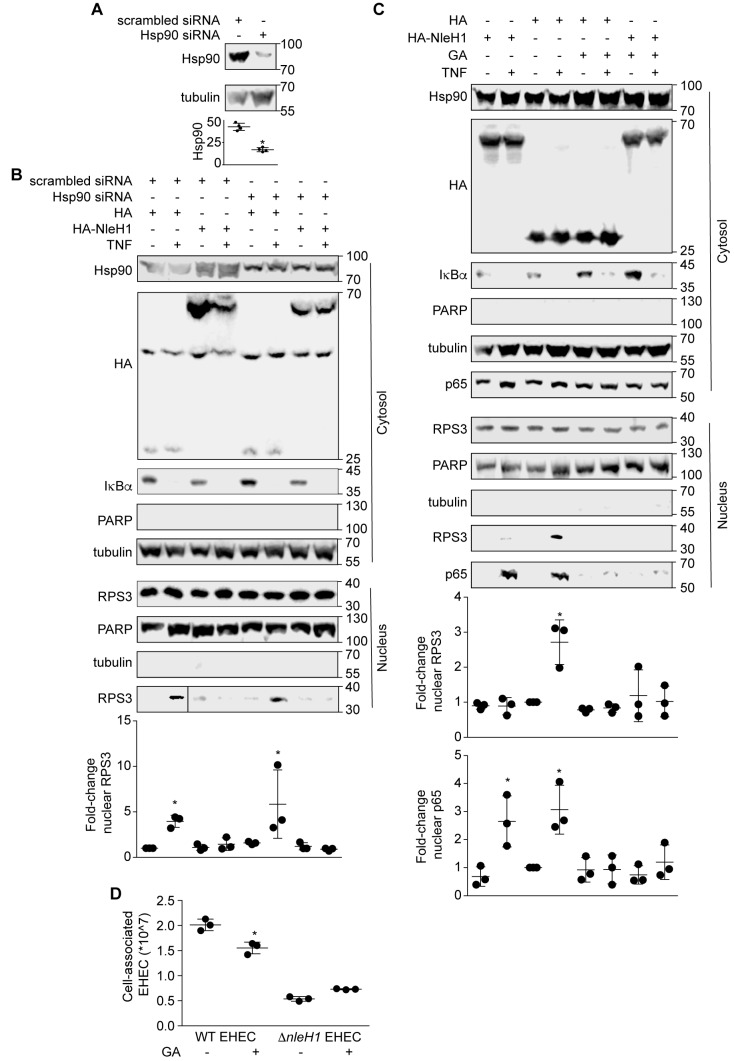
Geldanamycin (GA) inhibits RPS3 nuclear translocation. (**A**) Hsp90 knockdown with siRNAs. Asterisk indicates significantly different Hsp90 abundance, *t*-test. (**B**) Knockdown of Hsp90 does not alter RPS3 nuclear translocation. HEK293 cells were transfected with a pool of 4 Hsp90 siRNAs or with a nonspecific siRNA. After 24 h, cells were transfected with NleH1-HA or an HA epitope control. After 24 additional hours, cells were stimulated with TNF (50 ng/mL, 30’) and then lysed, separated into nuclear and cytosolic extracts, and used in immunoblotting experiments. Asterisk indicates significantly different nuclear RPS3 abundance, ANOVA. (**C**) GA inhibits RPS3 nuclear translocation. HEK293 cells were transfected with NleH1-HA or an HA epitope control. Cells were treated with 1 μM GA 5 h after transfection. After 20 h, cells were stimulated with TNF (50 ng/mL, 30’) and then lysed, separated into nuclear and cytosolic extracts, and used in immunoblotting experiments. Asterisks indicate significantly different nuclear RPS3 or p65 abundance, ANOVA. (**D**) Hsp90 inhibition reduces EHEC adherence. Caco-2 cells were treated with 1 μM GA for 20 h, and then were infected with EHEC strains at a multiplicity of infection (MOI) of 100. The number of bacteria adherent to Caco-2 cells was determined at 3 h post-infection by plating on LB agar plates. Asterisk indicates significantly different cell-associated bacteria, *t*-test.

To determine whether the interactions among Hsp90, NleH1 and IKKβ contribute to the ability of NleH1 to inhibit RPS3 nuclear translocation, we first established small interfering RNA (siRNA) knockdown conditions to reduce the steady-state levels of Hsp90 in HEK293 cells (Figure 3A). Hsp90 knockdown cells were transfected with either NleH1-HA or an HA epitope control plasmid. Cells were harvested after stimulation with TNF for 30 min (to induce RPS3 nuclear translocation), and then cell lysates were fractionated to separate cytosolic from nuclear proteins. RPS3 nuclear translocation induced by TNF was assessed by immunoblotting. TNF treatment induced a significant increase in RPS3 translocation to the nucleus (Figure 3B). NleH1-HA significantly inhibited RPS3 nuclear translocation. Knockdown of Hsp90 did not alter the ability of TNF to induce RPS3 nuclear translocation in cells (Figure 3B). Hsp90 knockdown also did not change the ability of NleH1-HA to inhibit RPS3 nuclear translocation with the TNF stimulus (Figure 3B).

We then used the Hsp90 inhibitor geldanamycin (GA) to determine whether Hsp90 activity is required for RPS3 nuclear translocation. Transfecting NleH1-HA significantly inhibited RPS3 nuclear translocation, but did not affect p65 nuclear translocation (Figure 3C). GA inhibited the ability of TNF to induce RPS3 and p65 nuclear translocation in cells (Figure 3C). GA did not alter the ability of NleH1-HA to block RPS3 nuclear translocation induced by TNF (Figure 3C).

To assess a potential role for NleH1/Hsp90 activities in EHEC virulence, we conducted adherence assays to quantify the number of bacteria associated with Caco-2 cells as a function of Hsp90 inhibition. We found that inhibiting Hsp90 with GA significantly reduced the number of adherent wild-type EHEC at 3 h post-infection (Figure 3D). An EHEC Δ*nleH1* mutant was unaffected by Hsp90 inhibition, reinforcing our other data that suggest a functional link between Hsp90 and NleH1. These data indicate a potential role for Hsp90 in EHEC virulence.

## 3. Discussion

Hsp90 is a highly conserved and abundant heat shock protein (HSP) involved in a myriad of cellular processes. The abundance of Hsp90 accounts for ~1–3% of the total cytosolic proteins [12]. It is required to facilitate protein folding, stabilize client proteins and protect proteins from ubiquitin-dependent proteasomal degradation [13]. Geldanamycin binds to the ATP-binding site of Hsp90 to abolish the interaction between Hsp90 and its client proteins [14]. GA abolishes TNF-induced IKKβ activation and IκBα degradation, thus blocking NF-κB activation [15]. GA treatment also causes proteasomal degradation of IKKβ [16], leading to altered migration of the IKK complex [11].

NleH1 and NleH2 share 84% amino acid sequence identity, but have differential abilities to regulate the NF-κB pathway [17]. NleH1, but not NleH2, inhibits the phosphorylation of RPS3 by IKKβ without affecting the kinetics of IκBα δεγραδατιον (degradation) [17]. However, when IKKβ is overexpressed, both NleH1 and NleH2 were shown to attenuate NF-κB activation [18]. Though our results indicated that both NleH1 and NleH2 interact with Hsp90, because only NleH1 inhibits RPS3 nuclear translocation, we thus only focused on NleH1 for these functional studies.

IKKβ is crucial for regulating NF-κB signaling and plays an important role in tumorigenesis, as evidenced by the fact that deletion of IKKβ in a colitis-associated cancer model leads to decreased tumor incidence [19] and that T-cell restricted constitutively active IKKβ improves tumor control [20]. Hsp90 activity is required for IKKβ biosynthesis and activation [16] and the formation of a Cdc37-Hsp90 complex is important for stabilizing the IKK complex [10].

Here we present evidence that Hsp90 also interacts with the EHEC T3SS effector NleH1. NleH1 only restricts a subset of the IKKβ substrates, most importantly RPS3 [4]. Hsp90 binds directly to RPS3 and this Hsp90-RPS3 interaction was previously shown to protect RPS3 from proteasome-dependent degradation [21]. Our studies now identify an additional role for Hsp90 in regulating RPS3 nuclear translocation. We determined that GA inhibited both TNF-mediated p65 nuclear translocation and RPS3 nuclear translocation, indicating that Hsp90 is an important cofactor in regulating host inflammatory responses to bacterial virulence proteins. We also identified a direct interaction between NleH1 and IKKβ and identified Hsp90 as a cofactor in this complex. Importantly, inhibiting Hsp90 had a significant impact on wild-type EHEC adherence to Caco-2 cells, but did not impact the adherence of EHEC Δ*nleH1*. Thus, there may be a functional link between Hsp90 and NleH1 that impacts EHEC virulence and warrants further investigation.

## 4. Materials and Methods

*Cloning, Chemicals, and Antibodies*. The strains and plasmids used in this study are listed in Table 1. All chemicals were used according to manufacturer’s recommendations and were obtained from Sigma, except for the following: Nickel-nitrilotriacetic acid (Ni-NTA) agarose beads (Qiagen, Hilden, Germany), Glutathione sepharose 4B GST-tagged protein purification resin (GE healthcare Life Sciences, Marlborough, MA, USA), Polyjet DNA In Vitro Transfection Reagent (SignaGen Laboratories, Rockville, MD, USA), TNF-α (Cell Signaling, Danvers, MA, USA). Antibodies were obtained from the following resources: anti-FLAG, anti-HA, Sigma; anti-IκBα, Cell Signaling; anti-β-tubulin, anti-β-actin, anti-His, Santa Cruz Biotechnology; anti-PARP, BD Transduction Laboratories; anti-RPS3, Proteintech Group.

*Protein purification.* IKKβ was cloned into pET28a, and NleH1, NleH2, and NleB1 were cloned into pET42a. Recombinant proteins were expressed in *E. coli* BL21(DE3) cells. Bacterial cultures were grown to an *OD*_600_ of 0.6, and isopropyl β-d-thiogalactopyranoside (IPTG) was added to a final concentration of 0.6 mM. After 4 h of additional growth, cells were pelleted using centrifugation, and lysed in 50 mM sodium phosphate, pH 8.0, supplemented with 0.5 mg/mL lysozyme and halt proteinase inhibitor (Thermo Fisher Scientific, Waltham, MA, USA). Lysates were incubated on ice for 30 min with occasional shaking, after which an equal volume of 50 mM sodium phosphate, pH 8.0, 1 M NaCl, 8 mM imidazole, 20% glycerol, 1% sarkosyl was added, followed by further incubation for 30 min. Lysates were sonicated, clarified by centrifugation, and the supernatants were applied to nickel-nitrilotriacetic acid beads (Qiagen) with end-to-end rotation for 2 h at 4 °C. After washing with 50 mM sodium phosphate, pH 8.0, 600 mM NaCl, 60 mM imidazole, 10% glycerol, proteins were eluted in 50 mM sodium phosphate, pH 8.0, 600 mM NaCl, 250 mM imidazole, 20% glycerol. Samples were resuspended in 2× SDS sample buffer, heated for 5 min at 95 °C, and analyzed using 10% SDS-PAGE.

*Cell culture and transfection.* HEK293 cells were maintained at 37 °C, 5% CO_2_ in DMEM supplemented with 10% fetal bovine serum (FBS) and penicillin-streptomycin (100 U/mL). Cells were seeded in a 6-well plates 18–24 h prior to transfection. Media was replaced with 1 mL complete DMEM per well 1 h prior to transfection. DNA was transfected into cells using Polyjet DNA transfection reagent (SignaGen Laboratories). After 24 h of incubation at 37 °C, the cells were harvested.

*Co-immunoprecipitation assay.* Transfected HEK293 cells were washed once using pre-chilled 1× PBS. Washed cells were scraped into pre-chilled 1× PBS, pooled, centrifuged at 12,000× *g* for 5 min. Supernatants were disposed, and cells were lysed in 50 mM Tris-HCl, pH 7.4, 0.15 mM NaCl, 1 mM EDTA, 1% Triton X-100, supplemented with halt protease inhibitor cocktail (Thermo Fisher). Samples were incubated on ice for 30 min, with occasional shaking, and lysates were collected by centrifugation at 12,000× *g* for 10 min at 4 °C. Anti-FLAG M2 Affinity Gel was incubated with cell lysates for 45 min at 4 °C. The mixture was pelleted by centrifugation at 7000× *g* for 45 s at 4 °C, and washed 3 times with 50 mM Tris-HCl, 250 mM NaCl, pH 7.4. Samples were resuspended in 2× SDS sample buffer, heated for 5 min at 95 °C, and analyzed using 10% SDS-PAGE.

*Pulldown assays.* GST-tagged NleH1 and NleH2 (10 µM) were immobilized on glutathione sepharose 4B beads (GE Healthcare) in 20 mM Tris-HCl, pH 7.9, 0.1 M NaCl, 5 mM MgCl_2_, 1 mM EDTA, 1 mM DTT, 0.2 mM PMSF, 20% glycerol, 0.1% Nonidet P-40, supplemented with 0.33 U/µL of DNase I and RNase A. After overnight incubation at 4 °C, the beads were incubated with His-tagged purified IKK-β proteins (10 µM) for 1 h at 4 °C. The beads were then washed 3 times with 20 mM Tris-HCl, pH 7.9, 1 M NaCl, 1 mM EDTA, 1 mM DTT, 0.2 mM PMSF, 20% glycerol, 0.1% Nonidet P-40. Proteins were eluted with 10 mM reduced glutathione and analyzed using 10% SDS-PAGE.

*Mass Spectrometry and Protein Identification*. For mass spectrometry analysis, 500 mg of HEK293T cells was collected and lysed in 50 mM Tris-HCl, pH 7.4, 0.15 mM NaCl, 1 mM EDTA, 1% Triton X-100, supplemented with halt protease inhibitor cocktail (Thermo Fisher) for 30 min on ice, with occasional shaking. Cell lysates were collected by centrifugation at 12,000× *g* for 10 min at 4 °C. Washed beads were incubated with cell lysates for 45 min at 4 °C. The mixture was pelleted by centrifugation at 7000× *g* for 45 s at 4 °C, and washed 3 times with 50 mM Tris-HCl, 250 mM NaCl, pH 7.4. The beads were resuspended in 2× SDS sample buffer, boiled and subjected to SDS-PAGE and an aliquot used for Western blotting analysis. The gel was stained with GelCode Blue Stain Reagent (Pierce Manufacturing, Bradenton, Fl, USA) overnight and destained with ddH_2_O. The stained bands of the gels were cut for in-gel tryptic digestion and introduced into an LTQ-FT tandem mass spectrometer (ThermoFinnigan). Mass spectra were acquired in the positive ion mode.

*RNA interference and transfection*. siRNAs targeting Hsp90, as well as a negative control siRNA, were obtained from Santa Cruz Biotechnology. Transient transfection of 25 pmol siRNA into HEK293 cells was performed using Lipofectamine RNAiMAX (Life Technologies, Carlsbad, CA, USA) according to the manufacturer’s instructions.

*Cell fractionation*. Nuclear and cytosolic protein extracts were obtained as described previously [22]. HEK293 cells were transfected with NleH1-HA and after 36 h, TNF-α was added at 50 ng/mL for 30 min to promote RPS3 nuclear translocation. Cells were harvested and resuspended in the buffer, nuclear and cytosolic protein extracts were prepared using the NE-PER nuclear and cytoplasmic extraction reagents (Thermo Fisher). Data were analyzed by Western blotting for nuclear RPS3. Poly(ADP-ribose) polymerase and β-tubulin were used to normalize the protein concentrations of nuclear and cytoplasmic fractions, respectively.

*EHEC adherence*. Caco-2 cells (ATCC HTB37) were grown at 37 °C/5% CO_2_ in DMEM (Gibco™, Carlsbad, CA, USA) and treated with 1 μM GA for 20 h. Caco-2 cells were infected with WT or Δ*nleH1* EHEC for 3 h at a multiplicity of infection (MOI) of 100. Non-adherent bacteria were removed from the cells by washing with PBS. Cells and adherent EHEC were scraped into 1% Triton X-100 in PBS, and serial dilutions were plated onto LB agar plates.

*Statistics*. Protein abundance was quantified using Li-COR Image Studio software. RPS3 and p65 nuclear abundance was analyzed statistically using either one-way analysis of variance (ANOVA) or *t*-tests. *p* values < 0.05 were considered significant.

## Figures and Tables

**Table 1:** Strains and Plasmids.

**Strains**		
*E. coli* BL21(DE3)	*E. coli* F^−^ *ompT hsdSB* (r_B_-m_B_-) *gal dcm* (DE3)	
BL21(DE3)/NleH1-pET42a	GST-EHEC NleH1	[5]
BL21(DE3)/NleH2-pET42a	GST-EHEC NleH2	[5]
BL21(DE3)/IKKβ pET28a	His-IKKβ	This study
EHEC EDL933	Wild-type *E. coli* O157:H7 isolate	CDC
EDL933 Δ*nleH1*	EHEC *nleH1* deletion	[5]
**Plasmids**		
HA	HA fusion expression	Clontech
NleH1-HA	HA fused to *E. coli* EDL933 NleH1	[5]
NleH2-HA	HA fused to *E. coli* EDL933 NleH2	[5]
Hsp90-HA	HA fused to Hsp90	This study
3× FLAG	FLAG expression	Sigma
3× FLAG-IKKβ	FLAG-IKKβ	[4]
3× FLAG-NleH1	FLAG-EHEC NleH1	This study
3× FLAG-NleH2	FLAG-EHEC NleH2	This study
pET42a	Bacterial GST fusion expression	Novagen
NleH1-pET42a	GST-EHEC NleH1	[5]
NleH2-pET42a	GST-EHEC NleH2	[5]
pET28a	Bacterial His6 fusion expression	Novagen
IKKβ pET28a	His-IKKβ	This study
